# Clinical and radiographic evaluation of immediate versus delayed loading of dental implants at anterior maxilla: a randomized controlled trial

**DOI:** 10.1186/s12903-026-09843-w

**Published:** 2026-10-02

**Authors:** Mohamed Gamal Hassanin Atia, Mohamed Said Hamed, Asmaa Yousri Abdallah, Ahmed Abdelmohsen Younis

**Affiliations:** 1 Oral and Maxillofacial Surgery Department, Faculty of Dentistry, Suez Canal University, Ismailia, Egypt; 2 Oral and Maxillofacial Radiology Department, Faculty of Dentistry, Suez Canal University, Ismailia, 41522 Egypt

**Keywords:** Partial edentulism of the maxilla, Dental implant loading, Tooth loss in the anterior maxilla

## Abstract

**Background:**

Immediate loading significantly improves oral health related quality of life (OHRQoL) as well as satisfaction of patients. Future trials will determine the role of immediate loading protocol in clinical scenarios with various amounts of available jaw bone using different numbers of implants to retain a fixed prosthetic restoration in partially edentulous maxilla. The aim of this study is to evaluate and compare the clinical stability and radiographic difference of osteointegration between immediate and delayed loaded dental implants in the anterior and premolar regions of the maxilla.

**Methods:**

Sixteen patients aged from 20 to 55 were divided into two equal groups. Group 1 consisted of 8 implants that were placed and loaded 6 months after placement, while group 2 consisted of 8 implants that were placed and immediately loaded with a provisional PMMA restoration; after 6 months, the provisional restoration was replaced by a fixed restoration. Each implant was clinically evaluated for implant stability using the Osstell device and radiographically evaluated for bone density around the implant using postoperative indirect standardized digital radiographs taken immediately after implant placement and after 3 and 6 months.

**Results:**

Both groups had a significant decrease in pain and swelling after surgery over time, but the immediate loading group had consistently lower values on day 1 (*p* = 0.001), day 3 (*p* = 0.023), and day 7 (*p* < 0.001). Moreover, the immediate loading group also had a significantly lower edema at all-time points (day 1: *p* = 0.005; day 3 and day 7: *p* < 0.001). Implant stability (ISQ) increased over time in both groups, while no statistically significant between-group difference was observed at baseline or at 6 months. Peri-implant bone density showed no significant differences between groups at any time point.

**Conclusions:**

Immediate implant loading was found to be a better alternative than delayed loading, as it provides comparable bone density and implant stability while significantly reducing postoperative pain and edema, consequently enhancing quality of life, aesthetic results, and early patient comfort without compromising radiographic performance.

**Trial registration:**

NCT07381283 (registration date: February 2, 2026).

**Graphical Abstract:**

**Figure:**
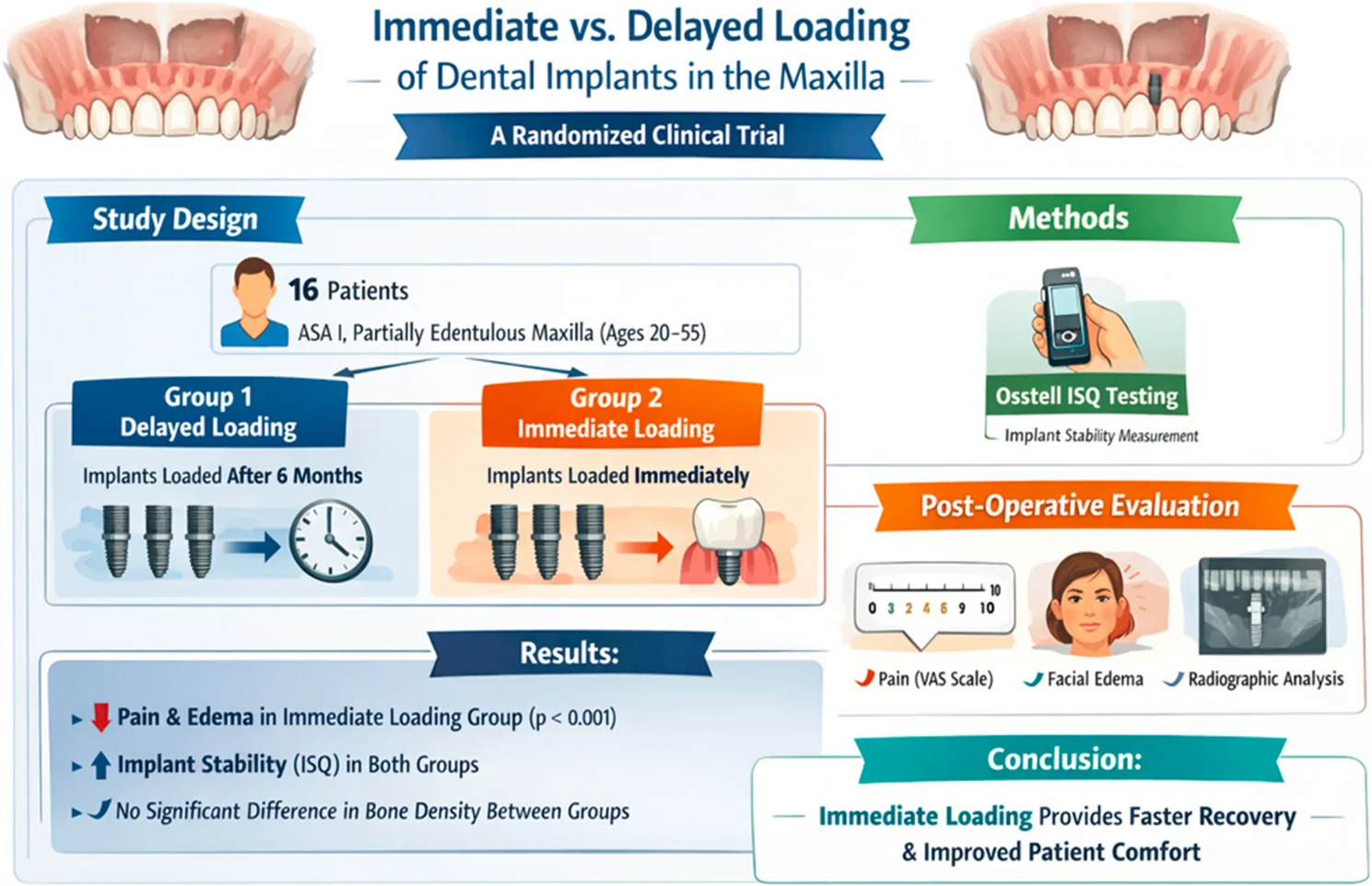

**Supplementary Information:**

The online version contains supplementary material available at https://doi.org/10.1186/s12903-026-09843-w.

## Background

Replacement of missing teeth with implant-supported single crowns has become a predictable and well-established treatment modality, providing high long-term survival rates together with favorable functional and esthetic outcomes [1]. In the anterior maxilla, implant therapy presents unique challenges because treatment success depends not only on osseointegration but also on preservation of the peri-implant hard and soft tissues to achieve optimal esthetics [2, 3]. Consequently, treatment protocols that shorten rehabilitation time while maintaining clinical success have attracted increasing interest [4]. 

Immediate loading, defined as the delivery of an implant-supported restoration within one week of implant placement, has gained popularity because it reduces treatment time, improves patient comfort, restores function and esthetics earlier, and minimizes the need for removable provisional prostheses. However, successful immediate loading depends on careful case selection and adequate primary implant stability to prevent excessive micromotion during the early healing period. Factors such as bone quality, implant design, insertion torque, occlusal loading, and surgical technique have all been identified as important determinants of successful osseointegration under immediate loading protocols [5, 6]. 

The biological success of immediate loading depends primarily on achieving sufficient primary implant stability to limit implant micromotion during the early healing phase [7]. Excessive micromotion at the implant–bone interface has been associated with fibrous tissue formation rather than direct bone apposition, thereby compromising osseointegration. Conversely, controlled functional loading under conditions of adequate primary stability may stimulate physiological bone remodeling through mechanotransduction, promoting maturation of the bone–implant interface and preservation of peri-implant tissues [7, 8]. Consequently, implant design, insertion torque, bone quality, and occlusal management are considered critical determinants of successful immediate loading protocols [9]. 

Despite the growing body of evidence supporting immediate loading, uncertainty remains regarding its application in single-tooth restorations within the anterior maxilla, where esthetic demands are high and bone quality is frequently less favorable. Previous systematic reviews and randomized clinical trials have generally demonstrated comparable implant survival rates between immediate and delayed loading protocols; however, differences remain regarding implant stability, marginal bone remodeling, and early clinical outcomes due to variations in implant systems, surgical protocols, and follow-up periods [10, 11]. 

Several randomized clinical trials and systematic reviews have demonstrated that immediate loading of dental implants can achieve high survival rates comparable to conventional delayed loading when appropriate clinical protocols, including adequate primary stability and careful case selection, are followed. Reported implant survival rates for immediately loaded implants frequently exceed 95%, with pooled analyses showing survival outcomes of approximately 97% in appropriately selected cases. Marginal bone level changes and implant stability have also been reported to be comparable between immediate and delayed loading protocols, although the available evidence remains heterogeneous, particularly regarding changes in implant stability during healing and radiographic bone remodeling in the esthetic zone of the anterior maxilla [12–14]. 

Although previous investigations have compared immediate and delayed loading protocols, relatively few randomized controlled trials have simultaneously evaluated implant stability, peri-implant bone density, and early postoperative outcomes in single-tooth implants placed in the anterior maxilla. Additional well-designed clinical studies are therefore required to clarify whether immediate loading provides clinical outcomes comparable to delayed loading while maintaining predictable osseointegration and patient recovery.

Accordingly, the aim of this randomized controlled trial was to compare the clinical and radiographic outcomes of immediate versus delayed loading of single dental implants placed in the anterior maxilla. Clinical outcomes included implant stability, postoperative pain, and edema, while radiographic evaluation assessed peri-implant bone density during the follow-up period.

## Methods

### Study design

**Fig. 1:**
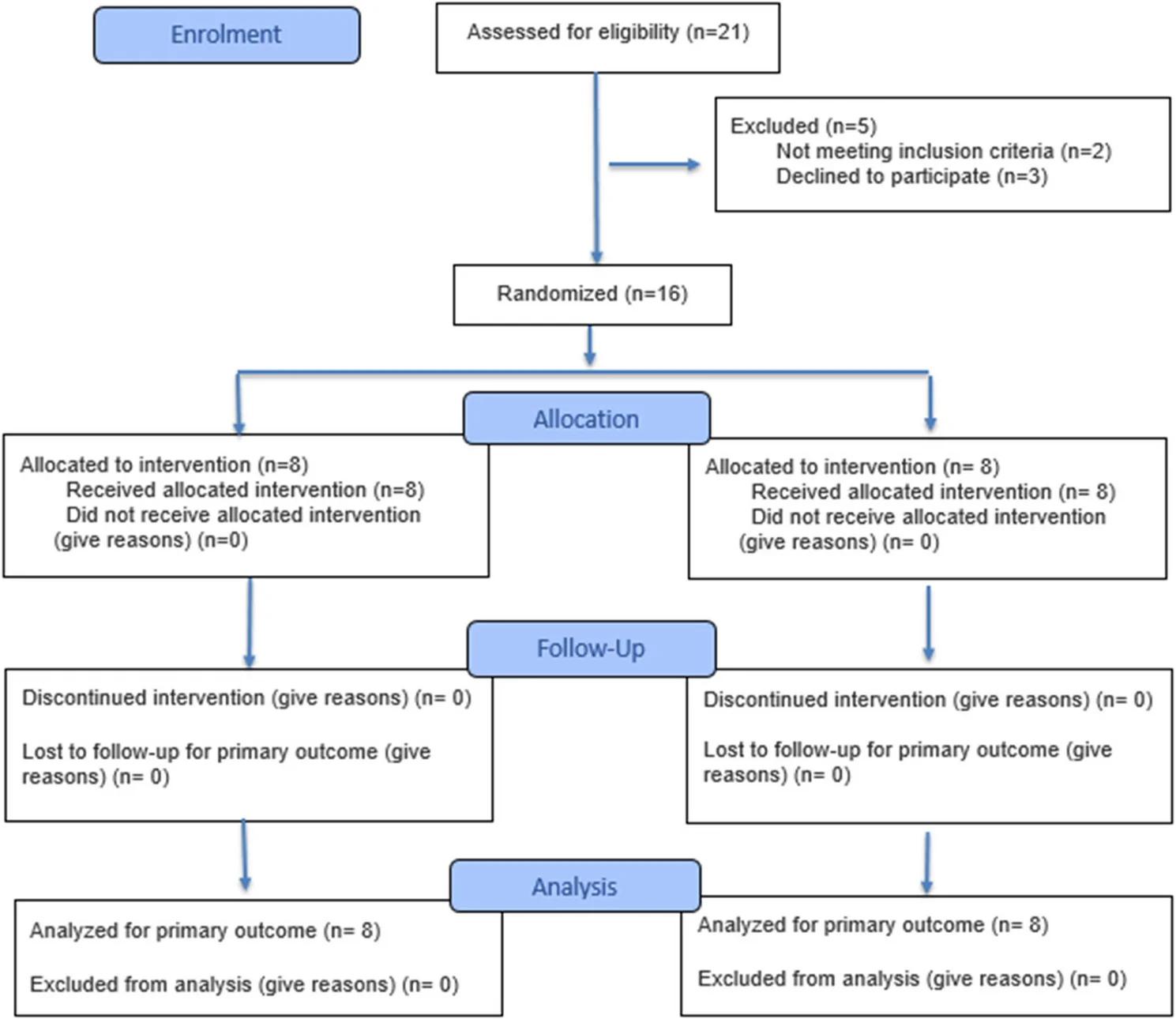
Flow diagram shows the CONSORT guidelines

This is an interventional, comparative, randomized clinical trial that was conducted at the Oral and Maxillofacial Surgery Department of the Faculty of Dentistry, Suez Canal University, Egypt. All participants were given an explanation of the study’s scope and approach at the initial assessment. The Suez Canal University Faculty of Dentistry’s Research Ethics Committee granted ethical approval (approval No. REC/706/2023). Written informed consent was obtained from all participants prior to enrollment. The clinical trial was carried out in compliance with the CONSORT criteria for clinical studies and was registered at ClinicalTrials.gov (NCT07381283) on February 2, 2026. Figure 1.

### Sample size calculation

The sample size calculation was performed a priori using G*Power software (version 3.1.9.6; Heinrich Heine University, Düsseldorf, Germany) [15]. The analysis was based on a repeated-measures ANOVA model with one between-subject factor (loading protocol) and one within-subject factor (follow-up time), corresponding to a group × time interaction effect. Implant stability quotient (ISQ), measured by resonance frequency analysis, was selected as the primary outcome because previous clinical investigation has demonstrated differences in ISQ progression between immediate and delayed loading protocols during osseointegration [16]. An effect size of Cohen’s f = 0.39 was assumed, corresponding to a partial eta squared (ηp²) of 0.13, calculated using the standard conversion formula described by Cohen. A two-sided alpha level of 0.05 and statistical power of 80% were applied. Based on these assumptions, the minimum required sample size was 16 participants (8 per group) [17, 18]. 

### Patient selection and eligibility criteria

The eligibility criteria were specified before participant recruitment:

#### Inclusion criteria

- Adult patients aged 20–55 years.
- American Society of Anesthesiologists (ASA I) with no significant systemic disease that could affect implant healing.
- Patients presenting with a single missing tooth in the anterior maxilla or maxillary premolar region, indicated for implant-supported rehabilitation.
- Adequate alveolar bone volume to allow implant placement with simultaneous guided bone regeneration, when required.
- Good oral hygiene and willingness to comply with the study protocol and follow-up visits.
- Patients who provided written informed consent to participate in the study.

#### Exclusion criteria

- Pregnant or lactating women.
- Current smokers or patients with parafunctional habits such as bruxism.
- Poor oral hygiene or inadequate plaque control.
- Active gingival or periodontal disease.
- Acute periapical infection, cysts, or other pathological lesions at the implant site.
- Patients unwilling or unable to attend the scheduled follow-up visits.

### Study groups and randomization

Participants were randomly allocated in a 1:1 ratio to either the delayed loading group (Group I) or the immediate loading group (Group II) using a computer-generated randomization sequence created with Randomizer.org. The allocation sequence was generated before participant enrollment by an investigator who was not involved in patient recruitment or clinical treatment. Allocation was concealed using sequentially numbered, opaque, sealed envelopes, which were opened only after implant placement to determine the assigned loading protocol.

Group I received delayed loading, with definitive loading performed 6 months after implant placement, whereas Group II received a provisional PMMA restoration within 5–7 days after implant placement according to the study protocol, followed by definitive restoration at 6 months.

### Pre-operative phase

#### History and clinical examination

Personal information such as name, age, gender, occupation, residence, and phone numbers were also recorded. Patients were also asked to provide their medical and dental history, including any health problems or behaviors that might affect the osseointegration process. Patients were assessed regarding oral hygiene, and teeth cleaning and polishing were performed with the help of the periodontology department before surgery began. Tooth distribution, side, and size measurements were also conducted. Along with taking x-rays for examination, patients were also given information regarding the surgical procedures they were to go through and any side effect that may occur, such as blood, pain, and swellings.

#### Radiographic examination

Before surgery, CBCT scans were taken using a Carestream Dental 3D SC system with CMOS sensor technology and a 75 μm pixel size. The scan lasted 11.9 s, with an effective exposure time of 3.2 s at 79 kVp, 8 mA, and a 0.5 mm focal spot, covering a 12 × 10 cm field of view that was adjusted using three laser guides. The images were processed with CS 3D Imaging Software; initial DICOM reconstruction took about two minutes, followed by additional reconstruction using On-Demand software. These scans were used to evaluate bone quality and dimensions for implant placement, clearly identify anatomical structures, and exclude patients with severe bone loss or nearby roots or carious teeth that required treatment.

#### Operative phase

**Fig. 2:**
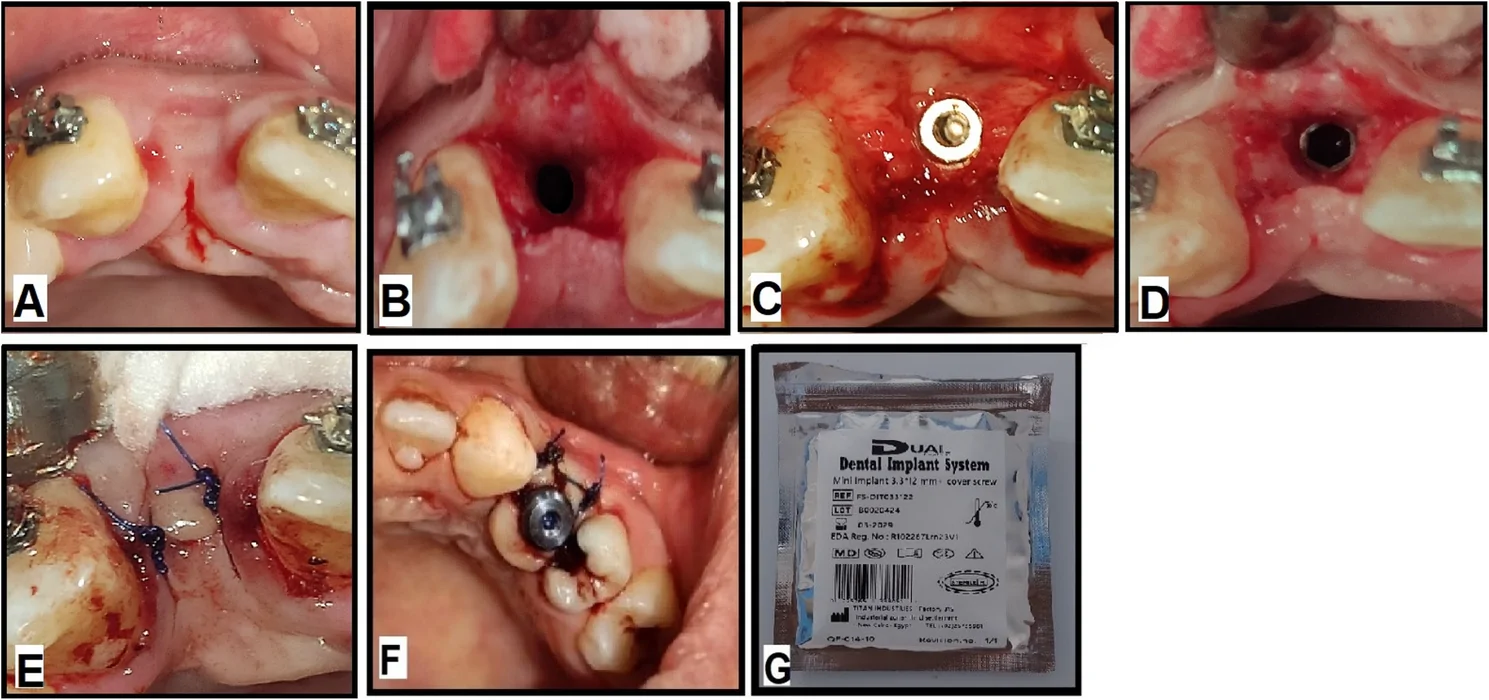
Representative intraoperative photographs demonstrating the implant placement procedure. **A** Preoperative clinical view (Control case); **B** prepared osteotomy site (Control case); **C** verification of implant angulation (Control case); **D** implant positioned at the planned depth (Control case); **E** wound closure in the delayed-loading group (Control case); and **F** healing abutment placement (Study case). All images are presented in standardized orientation

The surgical procedures shown in Fig. 2 were carried out following standard infection control measures to ensure patient safety. All surgeries were performed at the Oral and Maxillofacial Surgery Clinic, Faculty of Dentistry, Suez Canal University. All surgical procedures were performed under strict aseptic conditions by the same surgeon to minimize operator variability. Prior to surgery, patients were instructed to rinse with 15 ml of 0.1% chlorhexidine (Hexitol antiseptic mouth wash the Arab drug company ADCO, Cairo, Egypt) to reduce the oral microbial count. The patient’s perioral area was prepared using 10% povidone-iodine (Betadine the Nile Co. for pharma, Cairo, Egypt).

Using the infiltration technique, 4% articaine hydrochloride (Artinibsa 4% 1:100,000 solution for injection, Inibsa Dental SLU, Spain) was mixed with 1:100,000 epinephrine to provide local anesthetic.

Then incisions and mucoperiosteal flap elevation were done, in which a mid-crestal incision along the site of missing teeth and additional vertical releasing incisions were needed on the labial or buccal side of neighboring teeth on both sides. Then raising the full-thickness flap was done to expose the alveolar ridge.

The osteotomy site was further prepared using the initial drill under saline irrigation before implant placement. Drilling was performed carefully using a low-speed, high-torque handpiece connected to a surgical motor. The same drilling protocol was applied in all cases. Depending on the implant system used, drilling was completed in a sequential manner with continuous irrigation at a speed of 950–1100 rpm and a torque of 35 Ncm, until the planned osteotomy size was achieved.

The chosen implant package was opened and ready for implantation after all patients received DUAL dental implants (DUAL titanium dental implants, Titan Industries, Cairo, Egypt). Light, steady pressure was used to guide the implant into position. In order to put the implant 1–2 mm below bone level, it was first manually seated using an implant driver until two-thirds of its length was placed. A ratchet wrench was then used to finish the process.

Then, a smart peg attached to the implant was used to evaluate implant stability right after implantation, and the Osstell (ISQ) device’s probe provided the readings via a screen.

Finally, in the control group, wound closure was performed using figure-eight sutures to ensure complete stabilization of the surgical site, with no abutment placed immediately postoperatively; the abutment was delivered later during follow-up visits. In contrast, in the study group, healing abutments were immediately placed on the implants, and the surgical site was closed using simple interrupted polypropylene sutures to completely secure the wound until fabrication of the temporary crown.

**Fig. 3:**
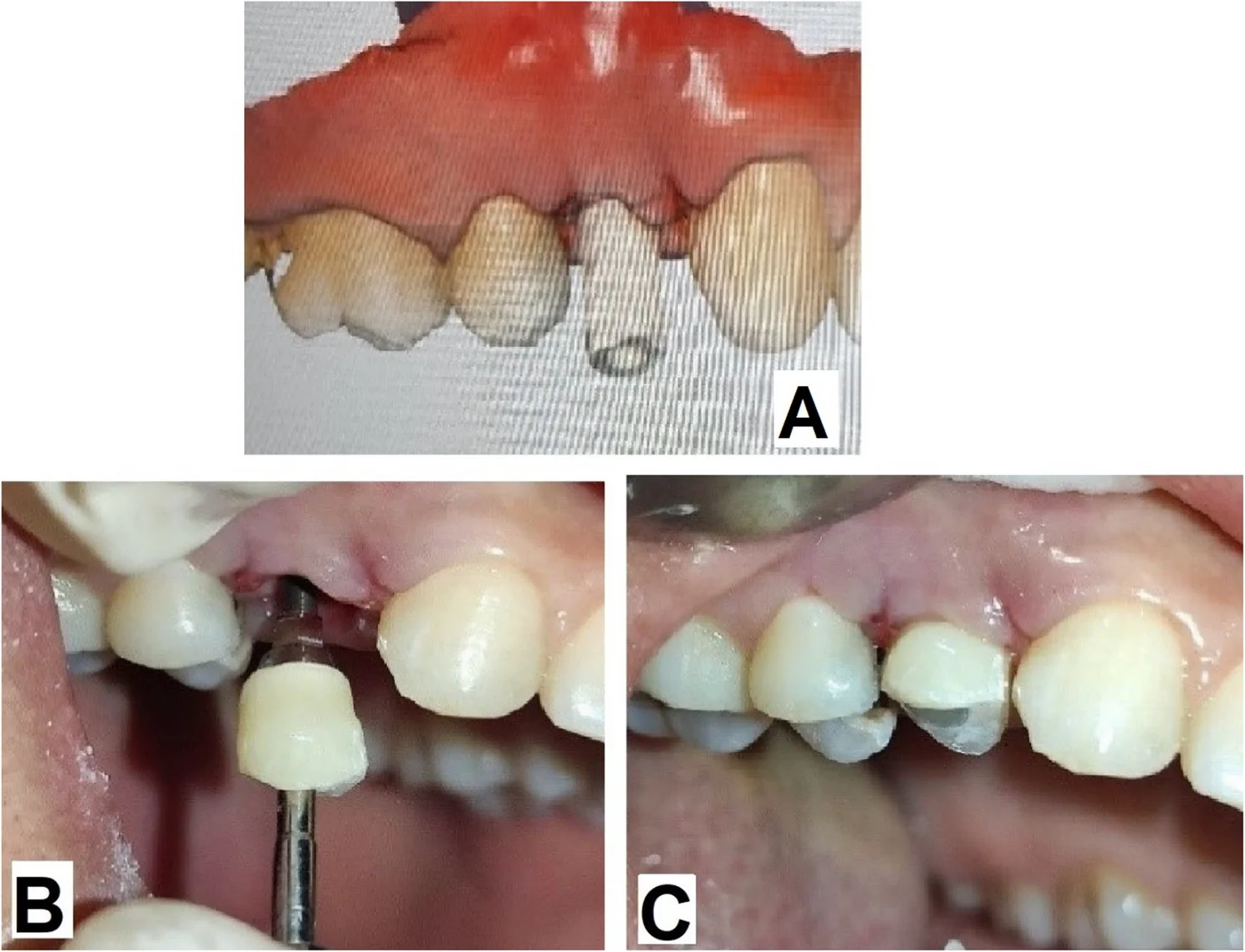
Provisional restoration; **A**. Frontal view during digital impression with scan body; **B**. PMMA provisional crown placement; **C**. occlusal reduction to maintain non-functional occlusion

Then, sutures and the healing abutment were removed within 5 to 7 days postoperatively, followed by the acquisition of a digital impression using a scan body. Subsequently, prosthetic temporization was performed, and the patients received a provisional restoration fabricated from PMMA. The provisional PMMA crowns were carefully adjusted to remain free of occlusal contacts in both maximum intercuspation and all eccentric mandibular movements. This non-functional occlusal scheme was adopted to minimize excessive loading and implant micromotion during the early healing phase while maintaining peri-implant soft tissue contour and esthetics. Figure 3.

### Post- operative phase

#### Post-operative instructions

During the recovery phase, patients were advised to practice proper dental hygiene and to prevent any trauma to the implant site. All patients were informed of the expected occurrence of facial edema, pain, and trismus. The sterile gauze pack was kept on the wound, and the patients were advised to bite for one hour. Also, patients were advised to avoid hot drinks, hot foods, hard foods, and smoking.

All patients in both groups were subjected to the following drugs after the surgery: Amoxicillin with clavulanic acid is available as 1 gm tablets every 12 h for 7 days. Acetaminophen is available as 500 mg tablets every 8 h. Chlorhexidine antiseptic mouthwash is available as 15 ml of 0.12%. Chlorhexidine mouthwash twice daily starting 8 h after surgery for 7 days postoperatively.

#### Post-operative evaluation

##### Visual analogue scale of pain (VAS)

Patients were asked to mark the degree of reported pain on a 10-cm horizontal line using a visual analog scale (VAS), where 0 (left side) denoted no pain and 10 (right side) denoted the most excruciating pain. The degree of pain was recorded after 1, 3, and 7 days of operation.

##### Visual edema scale

The degree of facial edema postoperatively was assessed using the “Visual Edema Scale” that is a clinical grading system based on the visual inspection and palpation of the swelling. The swelling was graded as “no edema” when the facial contours were normal, “low-grade edema” when the swelling was slight without any asymmetry or functional problems, “intermediate-grade edema” when the swelling was moderate with facial asymmetry and mild discomfort, and “high-grade edema” when the swelling was obvious with facial asymmetry and discomfort.

##### Implant stability

The stability of the implant was measured using the Osstell device (Osstell device (ISQ), Osstell AB, Gothenburg, 41101, Sweden), which is a non-invasive device that provides accurate and objective information regarding the extent of osseointegration. It shows the results on the spot and has a cord that connects the device to the probe. For the accuracy of the results, the smart peg was firmly attached to the implant. While using the device, it should be positioned squarely towards the center of the implant. It should have a maximum of 45 degrees of tilt towards the orthoradial line. Additionally, the probe tip should be 0.6 to 2.5 mm away from the implant surface. The stability of the implant is measured using the Implant Stability Quotient (ISQ), which ranges from 1 to 100. An ISQ of 70 and above indicates high stability, 60–69 indicates moderate stability, and less than 60 indicates low stability. The measurements were taken immediately after the implant was placed, and the final stability was checked six months later.

##### Radiographic evaluation

Post-operative radiographic evaluation utilized the Rinn extension cone paralleling (XCP) device with a modified silicone bite, alongside the KaVo Scan eXam™ One, to obtain digital periapical radiographs. The KaVo imaging plate, with specifications of 31 × 41 mm and 1034 × 1368 microns’ pixel size, operated wirelessly. A 16-inch cone was placed in line with the XCP film holder during the time of exposure. The Fona XDC machine was used as the x-ray machine.

**Fig. 4:**
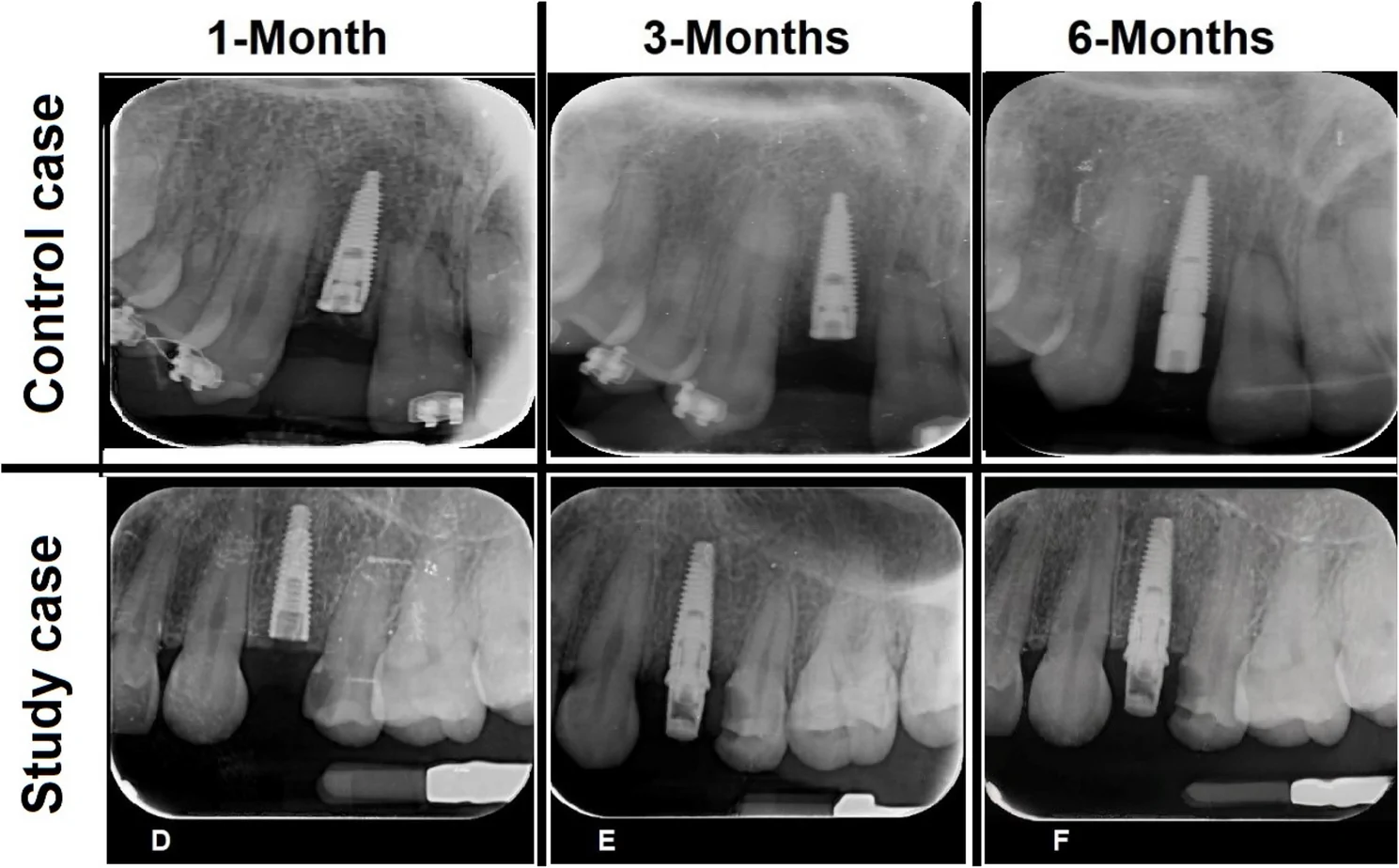
Standardized postoperative digital periapical radiographs demonstrating peri-implant bone density assessment in the control group (**A**–**C**) and study group (**D**–**F**). Images were obtained immediately after implant placement (baseline), at 3 months, and at 6 months, respectively

A single observer interpreted each radiograph to limit any potential sources of error. The radiographs were taken immediately after surgery, as well as three and six months post-operatively. Digital Image analysis software used was IDRISI Kilimanjaro (IDRISI Kilimanjaro software provided by Clark Labs, Clark University, Worcester, USA). Restoration of the images was done to allow densitometric measurements. The software was able to enhance the images to allow for the identification of the edges of the implants. The software provided innovative methods of tracking changes in bone density in two zones. The zones were the osseointegration zone (mean 1) and the surrounding bone zone (mean 2). Radiographic assessment was performed immediately postoperatively (baseline) and at 3 and 6 months postoperatively. Figure 4.

#### Permanent crown placement

**Fig. 5:**
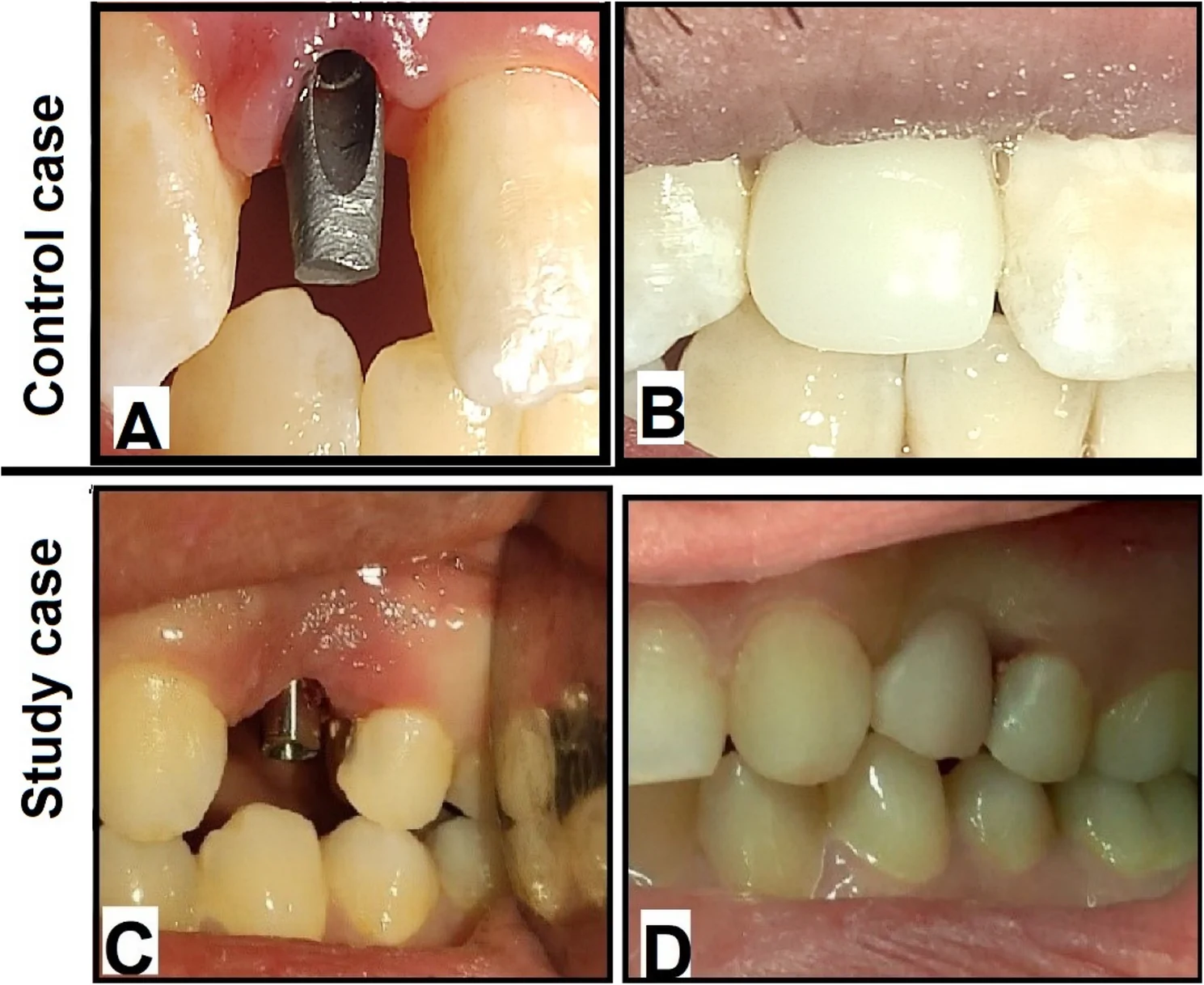
Representative definitive prosthetic rehabilitation at the 6-month follow-up. **A** control group: abutment verification before cementation; **B** control group: definitive crown in functional occlusion; **C** study group: abutment verification before cementation; and **D** study group: definitive crown in functional occlusion

In both groups, immediate loading (study group) and delayed loading (control group), a digital impression was obtained using a scan body six months after implant surgery. Following impression acquisition, definitive prosthetic rehabilitation was completed with the placement of the permanent crown. Figure 5.

#### Statistical analysis

SPSS software version 32.0.1 will used as the analysis tool with significance set at *P* < 0.05. All data will be calculated, tabulated, and analyzed using the appropriate tests. First, the Shapiro-Wilk normality test will be used to check the distribution of the sample data. For the data to be analyzed, the descriptive statistics will be given in the form of Mean ± SD, range (Max-Min). For the comparison of the time intervals in the groups, the one-way ANOVA test, or the Kruskal-Wallis test, will be used depending on the type of data. For the pairwise comparisons, the data will be analyzed using the Independent Student T-test, or the Mann-Whitney test, depending on the type of data.

## Results

### Demographic data

Regarding demographic and clinical characteristics, both groups show comparable results. The mean age for the delayed loading group (Group I) was 25.88 ± 2.42 years, compared with 24.13 ± 2.71 years for the immediate loading group (Group II), with no statistically significant difference (*p* = 0.224). Gender distribution in the immediate loading group was 100% female, with no males, whereas the delayed loading group had 12.5% male and 87.5% female individuals. 37.5% of the implants were on the right side of the maxilla, while 62.5% were on the left. Table 1.

**Table 1:** Demographic characteristics, implant site and side, and tooth distribution of both groups

Parameter		Delayed Loading	Immediate Loading	*p*-value
Age (years)		25.88 ± 2.42	24.13 ± 2.71	0.224
Gender	Male	1 (12.5%)	0 (0%)	0.334
	Female	7 (87.5%)	8 (100%)	
Implant site	Upper jaw	8 (100%)	8 (100%)	
Implant side	Right	3 (37.5%)	3 (37.5%)	0.990
	Left	5 (62.5%)	5 (62.5%)	
Tooth distribution	Central incisor	0 (0%)	1 (12.5%)	0.334
	Lateral incisor	2 (25%)	0 (0%)	
	Canine	1 (12.5%)	0 (0%)	
	1st premolar	3 (37.5%)	5 (62.5%)	
	2nd premolar	2 (25%)	2 (25%)	

### Clinical outcomes

#### Clinical assessment

The mean surgical duration was significantly longer in the immediate loading group (54.75 ± 4.21 min) compared with the control group (44.88 ± 7.41 min, *p* = 0.008). All cases in both groups showed normal mucosa at the implant site at different follow-ups. No case showed any postoperative infection (peri-implantitis). There was no redness, signs of inflammation, or fistula in any case in the study group or the control group.

#### Postoperative pain

Regarding pain score, it decreased gradually in both groups over time. On postoperative day one, the mean pain score of the control group was significantly higher (7.13 ± 0.78) compared to the mean pain score of the immediate loading group (5.75 ± 0.43); this difference was significant (*p* = 0.001). By day three, there was a reduction in mean pain scores in both groups, with (3.38 ± 0.48) in the control group and (2.75 ± 0.43) in the immediate loading group, which also shows a statistically significant difference (*p* = 0.023). By the seventh day, pain scores decreased to (2.63 ± 0.48) in the control group and decreased to (1.50 ± 0.50) in the immediate loading group, which again shows a significant difference (*p* < 0.001) between the two groups. Table 2.

**Table 2:** Comparison of pain scores between groups

Time Point	Control Group (Mean ± SD)	Immediate Loading (Mean ± SD)	*p*-value
Day 1	7.13 ± 0.78	5.75 ± 0.43	0.0011
Day 3	3.38 ± 0.48	2.75 ± 0.43	0.0233
Day 7	2.63 ± 0.48	1.50 ± 0.50	0.0008

#### Post-operative edema

One day postoperative, the mean edema score of the control group was consistently higher compared to the mean score obtained by the immediate loading group. The mean edema score of the control was 3.50 ± 0.53 compared to 2.62 ± 0.52 of the immediate loading group. The comparison is statistically significant (*p* = 0.005). By the third day of the study, the mean edema score of the immediate loading group was 1.50 ± 0.53 compared to 2.87 ± 0.35 of the control group. The comparison of the values is again significant (*p* < 0.001). On the seventh day of the study, the mean edema score of the control group was 2.62 ± 0.51 compared to 1.00 ± 0.00 of the immediate loading group, which is also statistically significant (*p* < 0.001). Table 3.

**Table 3:** Comparison of postoperative edema scores

Time Point	Control Group (Mean ± SD)	Immediate Loading Group (Mean ± SD)	*p*-value
Day 1	3.50 ± 0.53	2.62 ± 0.52	0.005
Day 3	2.87 ± 0.35	1.50 ± 0.53	< 0.001
Day 7	2.62 ± 0.51	1.00 ± 0.00	< 0.001

#### Implant stability

Both groups show a gradual increase in the mean ISQ values for implant stability. The control group showed an increase in mean ISQ values immediately post-operative from 53.83 ± 7.88 to 76.97 ± 10.06 after six months. In contrast, the mean ISQ values in the immediate loading group showed an increase from 60.19 ± 9.63 immediately post-operative to 85.50 ± 8.02 after six months. Overall, there was no significant difference between the two groups; however, the means were consistently higher in the immediate loading group. Table 4.

**Table 4:** Comparison of the mean ISQ values for implant stability

Time Point	Control Group (Mean ± SD)	Immediate Loading Group (Mean ± SD)	*p*-value
Immediate	53.83 ± 7.88	60.19 ± 9.63	0.198
6 Months	76.97 ± 10.06	85.50 ± 8.02	0.101

#### Relative bone density

Immediately postoperative, the mean bone density value of the control group was 58.86 ± 17.85, compared to 51.53 ± 12.25 in the immediate loading group, which was not statistically significant (*p* = 0.354). At 3 months post-operative, the mean bone density value of the control group was 69.43 ± 15.75, compared to 61.39 ± 14.71 in the immediate loading group, which was also not statistically significant (*p* = 0.309). At 6 months post-operative, the mean bone density value of the control group was 88.05 ± 27.99, compared to 83.22 ± 21.18 in the immediate loading group, which was also not statistically significant (*p* = 0.702). Although the delayed loading group demonstrated numerically higher mean bone density values throughout the follow-up period, the differences between the two groups were not statistically significant. Table 5.

**Table 5:** Comparison of the mean bone density values between groups

Time Point	Control Group (Mean ± SD)	Immediate Loading Group (Mean ± SD)	*p*-value
Immediate	58.86 ± 17.85	51.53 ± 12.25	0.354
3-months	69.43 ± 15.75	61.39 ± 14.71	0.309
6-months	88.05 ± 27.99	83.22 ± 21.18	0.702

## Discussion

The present randomized controlled trial compared the clinical and radiographic outcomes of immediate and delayed loading protocols for single dental implants placed in the anterior maxilla. Clinical outcomes included implant stability, postoperative pain, and edema, while radiographic evaluation assessed peri-implant bone density during the healing period. The principal findings were that implant stability and peri-implant bone density did not differ significantly between the two groups, whereas statistically significant differences were observed in postoperative pain and edema during the early postoperative period. Importantly, the provisional PMMA restoration in the immediate-loading group was delivered 5–7 days after implant placement and was intentionally kept out of occlusal contact. Therefore, the early differences in pain and edema should not be interpreted as direct effects of functional loading. Rather, they may reflect differences in early peri-implant healing conditions, including transmucosal versus submerged healing, soft-tissue management, and individual biological variability.

Immediate and delayed loading protocols have both been extensively investigated for implant-supported restorations, and accumulating evidence indicates that, when appropriate case selection and adequate primary implant stability are achieved, both approaches can provide predictable clinical outcomes with high implant survival rates. Several studies have demonstrated comparable implant survival, implant stability, and peri-implant bone preservation between the two loading protocols, although variations in patient selection, implant systems, surgical techniques, and outcome measures have resulted in some inconsistencies across studies [19, 20]. 

In agreement with our study, recent studies recruited healthy adults between 20 and 50 years of age to undergo the immediate loading of implants in the maxillary anterior zone. The exclusion criteria were pregnancy, smoking more than 10 cigarettes a day, systemic diseases that affect bone healing, poor oral hygiene, and active periodontal diseases [21, 22]. 

In this study, it was observed that there was no significant difference between the two groups in terms of age, gender, implant side, and distribution of teeth. In both groups, the implants were placed in the anterior maxilla; although the number of tooth sites differed slightly between groups, this difference was not statistically significant.

In line with our study, postoperative pain was monitored for patients using the Visual Analogue Scale (VAS), which measured the pain of patients and compared it precisely for different treatments and at different times [23]. In contrast, another study used the Short-Form McGill Pain Questionnaire instead of the Visual Analogue Scale (VAS) [24]. 

Postoperative pain decreased progressively in both groups during the first postoperative week, but the immediate-loading group reported significantly lower pain scores on days 1, 3, and 7. However, because the provisional PMMA restorations were delivered 5–7 days after implant placement and kept free of occlusal contacts, these findings cannot be attributed to functional immediate loading. The observed differences may instead reflect variations in the early peri-implant healing environment, including submerged versus transmucosal healing, soft-tissue management, and individual biological variability.

The available literature concerning postoperative pain in relation to implant loading protocols is also inconsistent. Vercruyssen et al. reported no significant difference in postoperative pain between immediately and conventionally loaded implants placed in the edentulous maxilla, whereas Mundt et al. reported greater postoperative pain following immediate loading [25, 26]. Differences among these studies may be related to variations in implant location, surgical approach, patient characteristics, loading protocol, prosthetic design, and pain assessment methods.

In the context of the present study, the lower pain scores in the immediate-loading group should be interpreted as an observation of early postoperative recovery rather than evidence of a direct benefit of functional loading. Notably, significant differences on days 1 and 3 occurred before provisionalization, and the provisional restoration was subsequently maintained in non-functional occlusion. Therefore, the study cannot establish a causal relationship between functional immediate loading and reduced postoperative pain.

The present study was consistent with a recent randomized trial that used a clinical edema scale, allowing the classification of patients according to the degree of swelling [27]. In contrast, another study measured edema by measuring the distance from the corner of the eye to the jawline and from the ear tragus to the mouth corner [28]. 

Regarding postoperative swelling, our results showed that there was a significant difference, in which the immediate loading group had lower edema scores at all-time intervals. On Day 1, we found that the edema scores were considerably higher for the control group than for the study group (*p* = 0.005). On Day 3, we also noted that the study group performed better than the control group in terms of reduced postoperative edema and a statistically significant difference (*p* < 0.001). On Day 7, it was noted that postoperative edema was almost resolved for the study group, while the control group still showed considerable swelling, with a statistically significant difference (*p* < 0.001).

As with pain, the early differences in postoperative edema cannot be attributed to functional occlusal loading, as meaningful loading had not occurred during the assessment period. Instead, these differences may reflect variations in the soft-tissue healing configuration, with submerged healing in the delayed-loading group versus transmucosal healing around a healing abutment in the immediate-loading group. Differences in flap management, tissue exposure, wound closure, and local tissue adaptation may therefore have contributed to the observed edema pattern.

Previous studies have reported varying postoperative swelling outcomes associated with different loading protocols. Zembić et al. and Gjelvold et al. reported favorable clinical outcomes associated with immediate loading, whereas Mundt et al. observed greater postoperative edema following immediate loading of mandibular implants [29, 30]. The discrepancy among these findings highlights the influence of factors other than the nominal loading category, including implant location, surgical technique, prosthetic configuration, soft-tissue management, patient-related factors, and the timing and method of edema assessment.

In line with our study, a study employed Osstell to measure implant stability (ISQ) during sinus floor elevation [31]. Conversely, another study utilized periotest M in a randomized controlled trial to assess primary implant stability [32]. 

Implant stability increased over time in both groups. In the delayed-loading group, mean ISQ increased from 53.83 ± 7.88 immediately after implant placement to 76.97 ± 10.06 at 6 months, whereas in the immediate-loading group, mean ISQ increased from 60.19 ± 9.63 to 85.50 ± 8.02 during the same period. These findings reflect the progressive increase in implant stability that occurs during healing and osseointegration.

Previous studies have reported comparable patterns. Granić et al. observed an increase in ISQ over time in both immediate- and delayed-loading protocols, consistent with the transition from mechanical to biological stability [33]. Similarly, Kim and Yoon reported no statistically significant differences between immediate and delayed loading with respect to implant-related clinical outcomes in their study, while Romanos and Nentwig also reported comparable outcomes between loading protocols during long-term follow-up [34, 35]. In contrast, Zhou et al. reported significantly higher ISQ values in an immediate-loading group, suggesting differences in the trajectory of osseointegration between protocols [36]. Such discrepancies may be explained by differences in implant systems, treatment sites, loading definitions, primary stability, patient selection, and follow-up periods.

Implant stability is a dynamic process involving primary mechanical stability immediately after implant placement and secondary biological stability through osseointegration. Primary stability is influenced by bone quality, implant macrodesign, surgical technique, and insertion parameters, whereas secondary stability develops progressively through new bone formation at the implant–bone interface. Therefore, adequate primary stability is essential for immediate loading protocols until biological integration provides long-term stability [37]. 

An additional factor that may influence the success of immediate loading is the patient’s bite force capacity. During the early healing phase, excessive occlusal loading may induce implant micromotion beyond the threshold compatible with successful osseointegration, potentially increasing the risk of fibrous tissue formation and implant failure [38]. Because maximum bite force varies considerably among individuals, patients with lower functional bite force may expose immediately loaded implants to reduced mechanical stresses during early healing, although direct clinical evidence linking bite force magnitude to implant survival remains limited [39, 40]. Therefore, careful patient selection, evaluation of parafunctional habits, achievement of adequate primary implant stability, and meticulous occlusal adjustment of the provisional restoration remain fundamental principles of immediate loading protocols [41, 42]. These biomechanical considerations may partly explain the predictable outcomes reported for immediate loading when strict case selection criteria are followed.

Controlled functional loading has been suggested to positively influence peri-implant bone remodeling by providing physiological mechanical stimulation that promotes bone formation and maturation around the implant. According to the principles of mechanotransduction and Wolff’s law, appropriate functional loading may enhance the adaptive response of peri-implant bone, potentially contributing to improved secondary stability and maintenance of crestal bone levels [43, 44]. Immediate loading protocols, when performed in carefully selected patients with adequate primary implant stability and controlled occlusal conditions, have demonstrated clinical outcomes comparable to conventional loading protocols, including similar marginal bone level changes in appropriately managed cases [45, 46]. However, because marginal crestal bone loss was not directly evaluated in the present study, these potential biological advantages should be interpreted cautiously and require confirmation through future randomized clinical trials.

In line with our study, a recent study uses periapical radiographs as a vital diagnostic tool due to their benefits, such as low magnification, geometric accuracy, cost-effectiveness, and minimal radiation exposure [47]. Conversely, another study utilized alternative radiographic techniques, like panoramic imaging, which provides lower magnification for evaluating bone density [48]. 

Our study is in line with previous studies that have used IDRISI Kilimanjaro for precise radiographic bone density measurements; [49] however, other studies have used software like ImageJ for the same purpose [50]. 

Our findings regarding bone density revealed that there was no significant difference in bone density between the two types of loading at any follow-up period, although it increased significantly over time in both groups. These findings suggest that immediate loading does not adversely affect peri-implant bone density maturation in the anterior maxilla. However, the progressive increase observed in both groups is consistent with normal bone healing and maturation, and the lack of a significant between-group difference indicates comparable outcomes rather than greater bone preservation or superior remodeling with immediate loading.

In agreement with our results, Shibly et al. found no significant difference between immediate loading and control groups regarding bone density and bone preservation after one year (*p* > 0.5) [51]. This further supports our idea of no adverse effect of immediate loading on the maintenance of the bone level. Similar results were obtained by Crespi et al. (2008) [52], Kim and Yoon [34]., and Peñarrocha-Oltra et al. [53], who did not report significant bone loss between the delayed and immediate loading protocols. These findings indicate that the immediate-loading protocol was not associated with significantly different peri-implant bone-density measurements compared with the delayed-loading protocol. Therefore, our results support the comparability of the two loading protocols, rather than the superiority of either protocol, with respect to the radiographic bone-density outcome evaluated.

In contrast, Akoğlan et al. reported that the loading protocol significantly influenced peri-implant bone density. Although bone density increased progressively in all groups during the healing period, the early loading group demonstrated significantly greater peri-implant bone density after one year than both the immediate- and delayed-loading groups, suggesting that the timing of functional loading may influence the extent of peri-implant bone remodeling. The discrepancy between their findings and those of the present study may be attributed to differences in the loading protocols investigated (including an early loading group), implant location (posterior versus anterior maxilla), bone quality, radiographic assessment methods, and the longer follow-up period employed in their study. These methodological differences may have influenced the pattern and magnitude of peri-implant bone remodeling observed between the studies [54]. 

Taken together, the findings of the present study suggest that immediate loading can achieve clinical and radiographic outcomes comparable to delayed loading under the conditions investigated. Specifically, no statistically significant differences were identified between the groups in implant stability or peri-implant bone density during the 6-month follow-up period. These findings are consistent with the broader literature indicating that immediate loading can be successfully implemented in appropriately selected patients when adequate primary stability and appropriate surgical and prosthetic protocols are present [19, 20, 45, 46]. 

Within the limitations of the present randomized controlled trial, immediate and delayed loading of single dental implants in the anterior maxilla resulted in comparable implant stability and peri-implant bone-density outcomes over a 6-month follow-up period. The statistically lower pain and edema scores observed in the immediate-loading group represent differences in early postoperative recovery, but they should not be interpreted as evidence of an effect of functional immediate loading because functional occlusal loading had not yet occurred during the relevant assessment period.

The absence of statistically significant differences in ISQ and peri-implant bone density suggests that the immediate-loading protocol investigated in the present study did not adversely affect these outcomes compared with delayed loading. At the same time, the available data do not support claims that immediate loading provides superior implant stability or greater preservation of peri-implant bone.

Future multicenter randomized controlled trials with larger sample sizes and longer follow-up periods are needed to further clarify the comparative effectiveness of immediate and delayed loading in the anterior maxilla. Such studies should additionally evaluate marginal peri-implant bone loss, esthetic outcomes, patient-reported outcomes, functional occlusal loading, and implant survival to provide a more comprehensive assessment of the long-term clinical consequences of different loading protocols.

## Conclusions

Immediate and delayed loading of single dental implants in the anterior maxilla demonstrated comparable implant stability and peri-implant bone-density outcomes during the 6-month follow-up period. Although statistically significant differences in postoperative pain and edema were observed during the early postoperative period, these findings preceded functional occlusal loading and therefore cannot be interpreted as direct effects of functional loading. Within the limitations of this study, the findings support comparable clinical and radiographic outcomes between the two loading protocols in appropriately selected patients.

## Supplementary Information

Supplementary Material 1.

Supplementary Material 2.

## Data Availability

All data obtained and examined throughout this study are analyzed and provided within this manuscript.
