# Supplementary material for: Clinical and radiographic evaluation of immediate versus delayed loading of dental implants at anterior maxilla: a randomized controlled trial
Source: BMC Oral Health. 2026 Oct 2;26:1921. doi: 10.1186/s12903-026-09843-w (PMC13637352; doi:10.1186/s12903-026-09843-w)
Supplement: Supplementary file 2 [file 12903_2026_9843_MOESM2_ESM.docx]

**Clinical and Radiographic Evaluation of Immediate Versus Delayed Loading of Dental Implants at Anterior Maxilla:
A Randomized Controlled Trial**

A Thesis Protocol

Submitted for partial fulfillment of the requirements of

the Master Degree in Dental Science

in

**Oral and Maxillofacial Surgery**

Research code: **HR1**

Submitted By

**Mohamed Gamal Hassanin Atia**

B.D.S, Faculty of Dentistry, Future University in Egypt, 2017

Dentist at Ministry of Health and Population

**Supervisors:**

| **Title** | **Name** | **Job Title** | **University** | **Signature** |
| --- | --- | --- | --- | --- |
| Prof. | Mohamed Said Hamed | Professor of Oral and Maxillofacial Surgery, Faculty of Dentistry | Suez Canal University |  |
| Dr. | Asmaa Yousri abdallah | Assistant professor of Oral Radiology, Faculty of Dentistry | Suez Canal University |  |
| Dr. | Ahmed Abdelmohsen Younis | Lecturer in Oral and Maxillofacial Surgery, Faculty of Dentistry | Suez Canal University |  |

Faculty of Dentistry

Suez Canal University

**2023**

**“Thesis Research Protocol”**

| **Student Name:** | Mohamed Gamal Hassanin Atia | | **Student ID:** | | 29309231300414 |
| --- | --- | --- | --- | --- | --- |
|  |  | |  | |  |
| **Submission Date:** | 16/5/2023 | | | | |
|  |  | | | | |
| **Degree:** | ☒ | **M.Dsc.** ☐ **D.Dsc.** ☐ **PH.D.** | | | |
|  |  |  | |  |  |
| **Department:** | Oral and Maxillofacial Surgery | | | | |
|  |  | | | | |
| **Thesis Title in English:** | Clinical and Radiographic Evaluation of Immediate Loading of Dental Implant at Anterior Maxilla | | | | |
|  |  | | | | |
| **Thesis Title in Arabic:** | التقييم السريري والإشعاعي للتحميل الفوري لغرسات الأسنان في الجزء الأمامي بالفـك العلـوي | | | | |

**1. Abstract**

**Introduction:** Immediate loading significantly improves oral health-related quality of life OHRQoL as well as satisfaction of patients. Future trials will determine the role of immediate loading protocol in clinical scenarios with various amounts of available jaw bone using different numbers of implants to retain a fixed prosthetic restoration in partially edentulous maxilla. **Aim:** The aim of this study is to evaluate and compare the clinical stability and radiographic difference of osteointegration between immediate and delayed loaded dental implants in the anterior and premolar regions of maxilla. **Methodology:** This clinical study will be divided equally and randomly by www.Randomizer.org into two groups; Group 1: 8 implants will be placed and loaded 4 months after placement (as control group) Group 2: 8 implants will be placed and immediately loaded by provisional restoration PMMA (as study group), and after 4 months the provisional restoration will be replaced by fixed restoration. Planning of implant insertion will be done by preoperative cone beam computerized tomogram (CBCT). Each implant will be clinically evaluated for implant stability using OSTELL Device and radiographically for bone density around the implant by postoperative indirect Standardized digital radiographs immediately after implant placement, after 3 and 6 months.

**2. Introduction and Background**

Rehabilitation of anterior maxilla is a challenging surgical and prosthetic procedure because of the anatomy (sinuses) and bone structure (thin cortex) surrounding a loose medulla with low trabecular density. Different options have been proposed in the literature as removable partial dentures, implant supported removable partial dentures, and implant-supported fixed partial dentures**. ( Mertens et al 2011)**

Implant-supported fixed dentures are considered a predictable partial edentulous maxilla treatment. This approach provides the closest feeling to natural dentition and improves patient confidence resulting in a high degree of satisfaction. Mertens et al reported an implant survival rate of 99% at an 8-year follow-up when treating completely edentulous maxilla **(Gallucci GO, et al 2018)**

patients with implants supporting screw-retained partial prostheses. According to Schwarz, this treatment choice demonstrates significant better results than implant-supported removal prostheses and is associated with lower post-loading implant loss rates. **(Schwarz F, et al 2016)**

The type of loading protocol (immediate, early, and conventional loading) is reported not to influence the implant and prosthesis survival rates; thus, all loading protocols can be recommended for the treatment of partial edentulous patients. **(Bergkvist G, et al 2009)**

Consequently, immediate loading protocols become the treatment of choice for patients as it reduces treatment time and increases patient comfort and satisfaction. Loading is considered as immediate if it takes place less than 1 week after implant placement. **(Bergkvist G, et al 2009)**

Immediate loading of dental implants is a contemporary treatment concept that contributes to success, provided important requirements are followed. Roccuzzo and Markovic´ demonstrated encouraging results with immediate loading of splinted and unsplinted implants in anatomical regions where bone quality was considered deficient. **(Fischer K, et al 2008)**

The primary objective of the present randomized clinical trial was to compare changes among primary and secondary implant stability between immediate and early loaded dental implants placed in edentulous maxillas using 2 different methods (Ostell). **(Wong, A.H., et al 2007)**

For immediate and early loading the primary stability of implants is crucial for a successful osseointegration and it is suggested that in edentulous arch the primary stability of each implant must be controlled. **(Niedermaier R, 2017)**

The stability of dental implants is mostly assessed by insertion torque (IT) and resonance frequency analysis (RFA). IT measures the frictional pressure between the implant and bone as Ncm. RFA measures an absence of movement after surgical implant insertion represented as implant stability quotient (ISQ) on a scale of 1 to 100. **(Niedermaier R, 2017)**

Up to the knowledge upon search of literature; there is not enough studies that demonstrate the role of immediate loading protocol in clinical scenarios with various amounts of available jaw bone using different numbers of implants to retain a fixed prosthetic restoration in partially edentulous maxilla

**3. Research Q (RQ):**

In patients with teeth loss at upper anterior and premolar regions, what are the differences in osseointegration and clinical stability between immediate and delayed loaded dental implants?

**4. Research Hypothesis, Aim, Objectives & Expected Outcomes**

**a. Hypothesis**

**Null hypothesis**: There is no significant difference in osteointegration and clinical stability between immediate and delayed loaded dental implants.

**Alternative hypothesis**: There is a significant osteointegration and clinical stability difference between immediate and delayed loaded dental implants.

**b. Aim**

The aim of this study is to evaluate and compare between immediate and delayed loaded dental implants in the anterior and premolar regions of maxilla.

**c. Objectives**

1-Clinical assessment: by checking OSTELL device readings of implant stability immediate postoperative, and after 6 months.
2-Radiographical assessment: by evaluating of bone density around the implant using preoperative cone beam computerized tomogram (CBCT) radiograph and postoperative indirect standardized digital radiographs immediately after implant placement, after 3 and 6 months.

**d. Expected Outcomes**

Both immediate and delayed loading protocols of implants may demonstrate similar changes in implant stability.

**5. Research Design and Methods**

**I. Material:**

Materials that will be used in this study mentioned in table (1).

**Table (1):** Materials, composition, and manufacturer

| **Materials** | **Composition** | **Manufacturer** |
| --- | --- | --- |
| Dual dental implants | Titanium | Cairo, Cairo  governorate, Egypt |
| Surgical lancets | Carbon steel | Swann Morton, England |
| Local anesthesia | Articaine | Cairo, Cairo  governorate, Egypt |
| Provisional restorations | Polymethyl methacrylate (PMMA) |  |
| Egyprolene | polypropylene | Cairo, Cairo  governorate, Egypt |

**II. Methods:**

a) **Study design:** The design of this study will be interventional comparative clinical study.
b) **Study setting:** This study will be carried out on patients selected from those who were referred to the department of oral and maxillofacial surgery Faculty of Dentistry, Suez Canal University; and during the initial examination, the purpose and methods of the research will be explained to the patients, and written informed consent will be assigned by the patients (Attached Appendix I)
c) **Study population and samples:**

**Patient Selection:**

Patient will be selected based on eligibility criteria.

**Inclusion criteria: (Kwiatek et al., 2021)**

1- Patients with teeth loss at upper anterior and premolar regions.
2- Both genders.
3- Middle aged adults (from 20 years to 55 years).

**Exclusion criteria: (Kwiatek et al., 2021)**

1- Medically compromised patient (uncontrolled diabetes, radiotherapy, chemotherapy and hemophilia)
2- Patient with bad oral habit as bruxism, smoking.
3- Patient with bad oral hygiene.
4- Patient with gingival diseases.
5- Patient with periodontal diseases (with sever vertical or horizontal bone loss).
6- Teeth with periapical or lateral infection or lesions.

**Sample Grouping:**

This clinical study will be conducted on 16 implants, will be divided equally and randomly by [www.Randomizer.org](http://www.Randomizer.org) into 2 groups:

Group 1: 8 implants will be placed and loaded 4 months after placement (as control group)

Group 2: 8 implants will be placed and immediately loaded (as study group)

**Clinical Procedures:**

**1. Anesthetic protocol:**

All surgeries will be performed under local anesthesia.

**2. Incisions and mucoperiosteal flap elevation:**

Mid crestal incision along the site of missing teeth and additional vertical releasing incisions may needed on the buccal side of neighbouring teeth on both sides will be performed.

Raising the full thickness flap to expose the alveolar ridge.

**3. Osteotomy**:

Under saline cooling, initial drill will be used, preparing for implant placement site.

**4. Implant placement:**

Afterwards the implants will be placed at the prepared site. OSTELL device reading will be recorded if the implant achieved good primary stability then we will perform immediate loading.

**5. Wound Closure:**

The wound will be sutured with a suitable material (polypropylene)

**6. Prosthetic temporization**

In contrast to the control group, the study group will receive a provisional restoration PMMA (as study group), and after 4 months the provisional restoration will be replaced by fixed restoration.

**7. Postoperative Care:**

Amoxicillin with clavulanic acid will be prescribed post-operatively for seven days (after making sure of the absence of allergy to any drugs used). Patient will be instructed to use paracetamol three times daily for three days, chlorohexidine rinses twice a day for one week.

Also the patients will be instructed to consume soft foods for two weeks and given the post-surgery instructions, smoking is prohibited and cold packs are used first 24 hours to decrease swelling.

**The clinical evaluation will be made by:**

OSTELL device which is used to monitor the implant stability. The implant stability will be measured immediately after implant placement by using a smart beg attached to the implant, then the implant stability will be measured by OSTELL and after 6 months.

**The radiographic evaluation will be made by: (Gupta et al., 2015)**

**1-** **Cone beam computerized tomogram (CBCT):**

Pre-operative Cone beam computerized tomogram (CBCT) using (Carestream Dental, CS8200 3D SC, France) with CMOS sensor technology and 75μm pixel size. The scanning time is 11.9 seconds of pulsed exposure resulting in an effective exposure time of 3.2 seconds. The exposure parameters will be 79KVp, 8Ma, and a focal spot size 0.5mm to scan FOV (field of view) of 12cm×10 cm. FOV adjustment is guided by three laser light beams to centralize the area of interest within the scanning field. All images will be captured, analyzed processed and stored using CS 3D Imaging Software (Carestream Dental, France). The primary reconstruction time for DICOM data set is two minutes. Then, the raw DICOM data set images will be imported to the On-Demand software (Cybermed, Korea) for secondary reconstruction and image analysis.

Each patient will be evaluated for bone quality, vertical and mesio-distal distance dimension of the potential site for implant insertion .

Cone beam computerized tomogram (CBCT) evaluation will be performed to allow for a more comprehensive overall view and better interpretation of the anatomic structures. As well as the patients who reveal severe bone loss will be excluded, others with neighboring remaining roots or carious lesions will planned for treatment.

**2-Intra oral paralleling periapical direct digital radiographic procedure:**

Post-operative indirect Standardized digital radiographs will be achieved using KaVo Scan eXam™ One and the Rinn extension cone paralleling (XCP) periapical film holder. The KaVo Scan eXam™ One is an Intraoral digital imaging plate system (psp) system using Imaging plate which is a film-like thin, flexible, and wireless phosphorescent plate, which works as a wireless receptor.

Using imaging plate size 2 has an active surface area of 31 X 41 mm, 1034x1368 micron (pixel size) and image size 2.69 megabytes.

A long cone, (sixteen inch in length) will mounted to the x-ray tube and the plastic aiming ring of XCP film holder will fixed flush ended with the round end of the long cone. The imaging plate will be exposed to the Fona XDC digital intraoral Xray machine.

The exposure parameters will be considered fixed for all patients. Processing will start after the end of the exposure using Scan eXam™ One unit then the image appears on the screen.

The stored images of each patient will be interpreted by one examiner at two different times to decrease intra and inter observer errors and the mean of the two trials will be recorded.

Each patient will be radiographically assessed immediately post-operative, after 3 months and after 6 months.

**6. Statistical plan**.

**A. Sample size calculation:**

The sample size calculation was performed a priori using G*Power software (version 3.1.9.6; Heinrich Heine University, Düsseldorf, Germany) **(Faul et al., 2007).** The analysis was based on a repeated-measures ANOVA model with one between-subject factor (loading protocol) and one within-subject factor (follow-up time), corresponding to a group × time interaction effect. Implant stability quotient (ISQ), measured by resonance frequency analysis, was selected as the primary outcome because previous clinical investigation has demonstrated differences in ISQ progression between immediate and delayed loading protocols during osseointegration **(Bischof et al., 2004).** An effect size of Cohen’s f = 0.39 was assumed, corresponding to a partial eta squared (ηp²) of 0.13, calculated using the standard conversion formula described by Cohen. A two-sided alpha level of 0.05 and statistical power of 80% were applied. Based on these assumptions, the minimum required sample size was 16 participants (8 per group) **(Cohen, 1988; Kyonka, 2018).**

Figure 1. Graphical presentation of α, β, power (1-β) and total sample size.

**Table (2): Variables of study**

| **Variable** | **Symbol** | | **Denote** |
| --- | --- | --- | --- |
| **Group (A)** | **A_0_** | **Group-I: (A_0_) Control group** | |
|  | **A_1_** | **Group-II: (A_1_) Study group** | |
| **Time of Examination**  **(T)** | **T_1_** | Measurement immediately postoperative | |
|  | **T_2_** | Measurement after 3 months | |
|  | **T_3_** | Measurement after 6 months | |

**Table (3): Interaction of variables (n= 16)**

| **Variables** | | **Treatment Group (A)** | | **TOTAL**  **Sample size** |
| --- | --- | --- | --- | --- |
|  |  | **A_1_**  **(8)** | **A_0_**  **(8)** |  |
| **Time of investigation (T)** | **T_1_** | **A_1_ T_1_** | **A_0_ T_1_** | **16** |
|  | **T_2_** | **A_1_ T_2_** | **A_0_ T_2_** |  |
|  | **T_3_** | **A_1_ T_3_** | **A_0_ T_3_** |  |
| **Total sample size** | | **8** | **8** | **16** |

B. **Statistical analyses:**

The statistical analysis will be performed for comparison between study and treatment groups (A_1;_ A_0_) at different time of investigations (T_1_, T_2_, and T_3_). The data will be collected, checked, revised, and organized in tables and figures using Microsoft Excel 2016. Data will be subjected to outliers’ detections and normality statistical test to detect whether the data are parametric or non-parametric.

Data will be analyzed for descriptive statistically both graphical and numerical description. Inferential statistics for evaluating and comparing between two different treatments (A_1_, A_0_), and time of investigations (T_1_, T_2_, T_3_) will be performed by repeated measures analysis of variance (ANOVA) or corresponding nonparametric analyses at significance levels of 0.05. ANOVA will be followed by Duncan multiple range tests (DMRTs) to compare between treatment groups or corresponding posthoc for nonparametric data. Difference between study and control groups will be performed by independent t-test at 0.05 level. Data analyses will be carried out using computer software Statistical Package for Social Science SPSS (IBM-SPSS ver. 28.0 for Mac OS) (Knapp, 2017).

**7. Ethics consideration**

The present research will be conducted after the approval of the Research Ethics Committee (REC) of the faculty of Dentistry, Suez Canal University. It will be conducted on five to sixteen patients selected for sixteen delayed dental implants insertion. Ethical considerations regarding patient well-being and confidentiality will be undertaken by the researcher and an informed written consent will be signed by the subjects/ patients before commencing the study explaining all clinical examinations, procedures and follow up.

**8. Time Plan**

**Starting:** After approval of ethical committee and faculty council

**Ending:** After 12 months

| **Activity/Month** | **1** | **2** | **3** | **4** | **5** | **6** | **7** | **8** | **9** | **10** | **11** | **12** | **13** | **14** | **15** | **16** | **17** | **18** | **19** | **20** | **21** | **22** | **23** | **24** |
| --- | --- | --- | --- | --- | --- | --- | --- | --- | --- | --- | --- | --- | --- | --- | --- | --- | --- | --- | --- | --- | --- | --- | --- | --- |
| Patient Selection | **☒** | **☒** | **☐** | **☐** | **☐** | **☐** | **☐** | **☐** | **☐** | **☐** | **☐** | **☐** | **☐** | **☐** | **☐** | **☐** | **☐** | **☐** | **☐** | **☐** | **☐** | **☐** | **☐** | **☐** |
| Implant procedure | **☐** | **☐** | **☒** | **☒** | **☒** | **☒** | **☐** | **☐** | **☐** | **☐** | **☐** | **☐** | **☐** | **☐** | **☐** | **☐** | **☐** | **☐** | **☐** | **☐** | **☐** | **☐** | **☐** | **☐** |
| Follow up | **☐** | **☐** | **☐** | **☐** | **☐** | **☐** | **☒** | **☒** | **☒** | **☒** | **☒** | **☒** | **☐** | **☐** | **☐** | **☐** | **☐** | **☐** | **☐** | **☐** | **☐** | **☐** | **☐** | **☐** |

**9. Research Estimated Budget in** Egyptian Pound

| **Supplementary** | | | | **Publications** | **Total** |
| --- | --- | --- | --- | --- | --- |
| Material | Radiographic examinations | Immediate loading | Final prosthetic |  |  |
| Implants  and surgical additive materials | Preoperative panorama, Postoperative digital periapical radiographs | Provisional restorations,  Postoperative antibiotics and analgesics | Final abutment and PFM crown |  |  |
|  |  |  |  |  |  |
| 45000 | 6000 | 5000 | 20000 | 4000 | 80000 |

Sponsored by the researcher himself

**10. References**

Bergkvist G, Nilner K, Sahlholm S, Karlsson U, Lindh C. (2009) ‘Immediate loading of implants in the edentulous maxilla: Use of an interim fixed prosthesis followed by a permanent fixed prosthesis: A 32-month prospective radiological and clinical study’, Clinical Implant Dentistry and Related Research, 11(1), pp. 1–10. doi:10.1111/j.1708-8208.2008.00094.x.

Bergkvist G, Sahlholm S, Nilner K, Lindh C. (2004) ‘Implant-supported fixed prostheses in the edentulous maxilla. A 2-year clinical and radiological follow-up of treatment with non-submerged ITI Implants’, Clinical Oral Implants Research, 15(3), pp. 351–359. doi:10.1111/j.1600-0501.2004.01017.x.

Cohen, J. 1988. Statistical power analysis for the behavioral sciences. Hillsdale, New Jersey: Lawrence Erlbaum Associates.

Eliasziw M, Young SL, Woodbury MG, Fryday-Field K. (1994) ‘Statistical methodology for the concurrent assessment of INTERRATER and intrarater reliability: Using goniometric measurements as an example’, Physical Therapy, 74(8), pp. 777–788. doi:10.1093/ptj/74.8.777.

Fischer K, Stenberg T, Hedin M, Sennerby L (2008) Five-year results from a randomized, controlled trial on early and delayed loading of implants supporting full-arch prosthesis in the edentulous maxilla, Europe PMC. Available at: https://europepmc.org/article/MED/16634495 (Accessed: 02 July 2023).

Gallucci GO, Benic GI, Eckert SE, (2014) ‘Consensus statements and clinical recommendations for implant loading protocols’, The International Journal of Oral &amp;amp; Maxillofacial Implants, 29(Supplement), pp. 287–290. doi:10.11607/jomi.2013.g4.

Gallucci GO, Hamilton A, Zhou W, Buser D, Chen S (2018) ‘Implant placement and loading protocols in partially edentulous patients: A systematic review’, Clinical Oral Implants Research, 29(S16), pp. 106–134. doi:10.1111/clr.13276.

Kim HS, Cho HA, Kim YY, Shin H. (2018) Implant survival and patient satisfaction in completely edentulous patients with immediate placement of implants: A retrospective study, BMC oral health. Available at: https://www.ncbi.nlm.nih.gov/pmc/articles/PMC6299569/ (Accessed: 02 July 2023).

Knapp, Herschel. 2017. Introductory Statistics Using SPSS. Second edition. Los Angeles: SAGE.

Mertens C, Steveling HG (2011) Implant-supported fixed prostheses in the edentulous maxilla: 8-year prospective results, Clinical oral implants research. Available at: https://pubmed.ncbi.nlm.nih.gov/21087314/ (Accessed: 01 July 2023).

Niedermaier R, Stelzle F, Riemann M, Bolz W, Schuh P, Wachtel H. (2017) Implant‐supported immediately loaded fixed full‐arch dentures: Evaluation of implant survival rates in a case cohort of up to 7 years, clinical implant dentistry and related research, DeepDyve. Available at: https://www.deepdyve.com/lp/wiley/implant-supported-immediately-loaded-fixed-full-arch-dentures-hKzmbvTrzb (Accessed: 01 July 2023).

Okada, T., Kanai, T., Tachikawa, N., Munakata, M. and Kasugai, S. (2017). Histological and Histomorphometrical Determination of the Biogradation of β-Tricalcium Phosphate Granules in Maxillary Sinus Floor Augmentation: AProspectiveObservationalStudy. Implantdentistry, 26(2),275–283. doi:10.1097/ID.0000000000000577.

Papaspyridakos P, Chen CJ, Chuang SK, Weber HP. (2014) ‘Implant loading protocols for edentulous patients with fixed prostheses: A systematic review and meta-analysis’, The International Journal of Oral &amp;amp; Maxillofacial Implants, 29(Supplement), pp. 256–270. doi:10.11607/jomi.2014suppl.g4.3.

Schwarz F, Sanz-Mart ın I, Kern J-S, Taylor T, Schaer A, Wolfart S Sanz M (2016) ‘Loading protocols and implant supported restorations proposed for the rehabilitation of partially and fully edentulous jaws. Camlog Foundation Consensus report’, Clinical Oral Implants Research, 27(8), pp. 988–992. doi:10.1111/clr.12736.

Wong, A.H., Cheung, C.S. and McGrath, C. (2007) ‘Developing a short form of Oral Health Impact Profile (OHIP) for Dental Aesthetics: OHIP-aesthetic’, Community Dentistry and Oral Epidemiology, 35(1), pp. 64–72. doi:10.1111/j.1600-0528.2007.00330.x.

Faul F, Erdfelder E, Buchner A, Lang AG. G*Power 3: a flexible statistical power analysis program for the social, behavioral, and biomedical sciences. Behav Res Methods. 2007;39:175-191.

Bischof M, Nedir R, Szmukler-Moncler S, Bernard JP, Samson J. Implant stability measurement of delayed and immediately loaded implants during healing. Clin Oral Implants Res. 2004;15:529-539. doi:10.1111/j.1600-0501.2004.01042.x.

Kyonka EGE. Tutorial: small-N power analysis. Perspect Behav Sci. 2018;42(1):133-152. doi:10.1007/s40614-018-0167-4.

**11. Appendices :(informed consent )**

**Suez Canal University**

**Faculty of Dentistry**

**Research Ethics Committee (REC)**

**------------------------------------------------------------------------------------------------------------**

**----------------**

**Investigator Application Form**

1-Name of researcher: Mohamed gamal hassanin atia

2-Name of Department: Oral and Maxillofacial surgery

3-Adress of researcher: Ismaailia

a- Email: elgazalimohamedm@gmail.com
b- Phone number: 01112760284
c- Fax number: none

4- Name (s) of Co-investigator (s): Mohamed Said Hamed

5- Grade of protocol:

*M.D.Sc. (**√**)	*Ph.D. (	)	*Doctorate degree (D.D.Sc.) (	)	*Other	(	)

*Domestic	(	)	*Multi-Centre within Egypt	(	)	*International	(	)

6- Title of the research: Clinical and Radiographic Evaluation of Immediate Vs Early Loading of Dental Implant at Edentulous Maxilla (A Comparative Study)

7-Type of the research:

*Drug trial	(**√** )	*Surgical technique	(**√** )	*Investigative technique (**√** )

*Devise study	(	)	*Survey study	(	)	*Blood sampling	(	)*Review of old records	(	)

8-Subjects of research:

*Children	(˂ 18 years): (	)	*Adults (>18 years)	(**√**) * Vulnerable groups (no)

-1-

**Suez Canal University**

**Faculty of Dentistry**

**Research Ethics Committee (REC)**

------------------------------------------------------------------------------------------------------------------------------------

9-Request is being made to ***waive***(give-up) informed consent: Yes: (	)	No: (**√** )

10- The research is for the good of society: Yes: (**√**)	No: (	)

11-Study design:

a-Phase type	I: (	)	II: (	)	III: (	)

b-Randomization: Yes: (	)	No: (**√** )

c-Placebo: Yes: (	)	No: (	)

d-Genetic sampling: Yes: (	)	No: (	)

e-Other: Yes: (	)	No: (	)

12-Facilities for the research are available: Yes: (**√**)	No: (	)

13- List the risks of the study:

Postoperative edema

Postoperative infection

Allergic reaction

14- List the potential benefits, if any, to the subjects

Replacing unrestorable teeth in less treatment time with more durability

15-Are the risks reasonable to the potential benefits to the subjects, if any, or to the

knowledge to be gained? Yes: (**√**)	No: (	)

16-Privacy and confidentiality of subjects are assured	Yes: (**√**)	No: (	)

17-The subject of the research could quit at any time without penalty or loss of any benefits

to which they would otherwise be entitled	Yes: (**√**)	No: (	)

---------------------------------------------------------------------------------------------------------------------------

Signature of the principal investigator	Date:

-2-

**التقييم السريري والإشعاعي للتحميل الفوري لزراعة الأسنان في الجزء الأمامي بالفـك العلـوي**

خطة بحث مقدمة توطئة للإتمام الجزئي للحصول على

درجة الماجستيرفي علوم طب الأسنان

**في جراحة الفم والوجه والفكين**

كود البحث	**HR1**

مقدمة من

**محمد جمال حسانين عـطـية**

بكالوريوس طب وجراحة الفم والأسنان كلية طب الأسنان جامعة المستقبل 201**7**

طبيب أسنان بوزارة الصحة والسـكان

**المشرفين**

| **اللقب** | **الإسم** | **الوظيفة** | **الجامعة** | **التوقيع** |
| --- | --- | --- | --- | --- |
| أ.د. | محمد سعيد حامد | أستاذ جراحة الفم والوجه والفكين⸲ كلية طب الأسنان | جامعة قناة  السويس |  |
| د. | أسماء يسري عبد الله | أستاذ مساعد أشعة الفم⸲ كلية طب الأسنان | جامعة قناة  السويس |  |
| د. | أحمد عبد المحسن يونس | مدرس جراحة الفم والوجه والفكين⸲ كلية طب الأسنان | جامعة قناة  السويس |  |

كلية طب الأسنان

جامعة قناة السويس

**2023**

1) **الملخص العربي**:

التحميل الفوري يحسن بشكل كبير نوعية الحياة المرتبطة بصحة الفم OHRQoL وكذلك رضا المرضى. ستحدد التجارب المستقبلية دور بروتوكول التحميل الفوري في السيناريوهات السريرية مع كميات مختلفة من عظام الفك المتاحة باستخدام أعداد مختلفة من الغرسات للاحتفاظ بترميم اصطناعي ثابت في الفك العلوي الأمامي.

2) **المقدمة**:

تعد إعادة تأهيل الفك العلوي الأمامي إجراءً جراحيًا وتعويضيًا صعبًا بسبب طبيعة التشريح (الجيوب) والبنية العظمية (القشرة الرقيقة) المحيطة بالنخاع الرخو ذي الكثافة التربيقية المنخفضة. يوفر هذا النهج أقرب شعور للأسنان الطبيعية ويحسن ثقة المريض مما يؤدي إلى درجة عالية من الرضا.

3) **الهدف من البحث:**

الهدف من هذه الدراسة هو تقييم ومقارنة بين التحميل الفوري والتحميل المتأخر لغرسات الأسنان في الجزء الأمامي بالفـك العلـوي.

4) **أسئلة البحث:**

في المرضى الذين يعانون من فقدان الأسنان في الفك العلوي بالمناطق الأمامية والضواحك ، ما هو تأثير التحميل الفوري على الاندماج العظمي لغرسات الأسنان فيما يتعلق باستقرار الغرسة وكثافة العظام فور وضع الغرسة و بعد ثلاثة و ستة أشهر؟

5) **خطة البحث ومنهجية الدراسة:**

هذه الدراسة تتضمن ستة عشر غرسة لتعويض أسنان أمامية أو ضواحك مفقودة في الفك العلوي، سوف يتم تقسيم الغرسات الى مجموعتين:

مجموعة 1 : سيتم وضع **8** غرسات وتحميلها بعد 4 أشهر من وضعها (كمجموعة تحكم)

مجموعة 2 : سيتم وضع **8** غرسات وتحميلها على الفور (كمجموعة دراسة)

نتائج الاجراء سوف يتم تقييمها سريريا و شعاعيا لتقييم ثبات الغرسات و التحام العظم .

سيتم شرح كل الخطوات الخاصة بالبحث لكل المرضى المشاركين و سيتم الحصول على موافقة مكتوبة منهم على كافة خطوات البحث .

6) **الخطة الإحصائية:**

- تم حساب حجم العينة وفقا لإصدار برنامج 3.1.9.6 G * Power .

- ستعرض النتائج في جداول ورسوم بيانية حسب الاقتضاء .

- سيتم تقديم البيانات على أنها متوسط ± الانحراف المعياري .

- سيتم استخدام اختبار ANOVA للقياسات المتكررة لمقارنة البيانات .

- تعتبر القيمة الاحتمالية <0.05 ذات دلالة إحصائية .

7) **الجدول الزمني:**

| رقم المعيار | وصف المرحلة | التاريخ المتوقع للانتهاء من العمل | ملاحظات |
| --- | --- | --- | --- |
| 1 | الإعداد | شهران |  |
| 2 | شراء الخامات المساعدة للبحث | شهر |  |
| 3 | الجزء العملي | 6 أشهر |  |
| 4 | تجميع البيانات | شهر |  |
| 5 | التحليل الإحصائي | أسبوعين |  |
| 6 | تغيير النتائج | أسبوع |  |
| 7 | كتابة المراجعة الأدبية | أسبوع |  |
| 8 | كتابة طرق البحث | أسبوع |  |
| 9 | كتابة المناقشة | أسبوعين |  |
| 10 | تجميع الرسالة كاملة | اسبوع |  |

**8) المرفقات (الاستمارة المستنيرة):**

**جامعة قناة السويس**

**كلية طب الأسنان**

**لجنة أخلاقيات البحث العلمي نموذج الموافقة المستنيرة**

**لإجراء بحث طبي على مشارك متطوع**

اسم المشارك (المريض):........................................ النوع :..........................

السن:......................................... تاريخ الميلاد:........................................

1- عنوان البحث باللغة العربية: التقييم السريري والإشعاعي للتحميل الفوري لزراعة الأسنان في الجزء الأمامي بالفـك العلـوي

2- الخلفية العلمية (لموضوع البحث) والهدف من إجراء البحث: قياس تأثير التحميل الفوري مقابل التحميل المتأخر على غرسات الأسنان سريريا وبإجراء الفحوصات الإشعاعية الازمة.

3- ما سوف يتم اجراؤه بالتفصيل:

*- مدة البحث ومكانه: ستة أشهر – كلية طب الأسنان جامعة قناة السويس

*- عدد المشاركين في البحث: ( مرضي وأصحاء):عدد يتراوح بين 3-10

*- أسلوب اختيار المشاركين في البحث (خاصة في التجارب الإكلينيكية)

- المرضى الذين يعرفون ويوافقون على إتباع جميع إجراءات الدراسة والذين يقدمون موافقة كتابية وتتراوح أعمارهم بين 20 إلى 55 عاما ولديهم فراغات لأسنان علوية أمامية مخلوعة

*-تفاصيل خطوات البحث :

- تلقي جميع المرضى التخدير الموضعي
- وضع غرسة الأسنان ثم التحميل الفوري او التحميل المتأخر
- قياس الثبات الأولى السريري للغرسة و مقدار التئام العظم حول الغرسة شعاعيا مباشرةً بعد الزراعة
- قياس الثبات السريري للغرسة و مقدار التئام العظم حول الغرسة شعاعيا بعد ثلاثة أشهر من الزراعة
- قياس الثبات السريري للغرسة و مقدار التئام العظم حول الغرسة شعاعيا بعد ستة أشهر من الزراعة

4- الفوائد المتوقعة من البحث (الفوائد المباشرة والغير مباشر): يعمل التحميل الفوري على تحسين جودة الحياة المرتبطة بصحة الفم لدى المرضى ويقلل بشكل كبير من الألم الجسدي والإعاقة النفسية.

5-المخاطر المحتمل حدوثها من اجراء البحث: قد تحدث مضاعفات أثناء الجراحة أو تتطور خلال فترة ما بعد الجراحة. قد تشمل مضاعفات ما بعد الجراحة نزف أو تورم نتيجة الجراحة أو فشل الغرسة

6- التعويضات في حالة حدوث مخاطر: يتم التكفل الكامل بعلاج المشارك بالشكل المناسب بكل الطرق العلاجية المتاحة ومتابعة المشارك بشكل دوري حتى ضمان الشفاء وعدم حدوث مضاعفات

7-البدائل المتاحة: في حالة رفضك الاشتراك في هذا البحث: (ستتلقى علاجك المعتاد والذي يعتبر إما خلع وتعويض السن المفقود بتركيبات ثابتة أو متحركة).

8- سرية معلوماتك: سوف تعامل معلوماتك بسريه كامله ولن يطلع عليها سوي الباحث الرئيسي. بعد انتهاء الدراسة سيتم ابلاغك بالنتائج وأي نتائج خاصه تتعلق بحالتك الصحية. -1-

9- حقك في الأنسحاب: من حقك الأنسحاب من البحث في أي وقت دون ابداء أسباب ودون أي عواقب سلبيه عليك.

10- في حالة أخذ أي عينه: فسوف لا تستخدم في أي بحث آخر.

11- عند وجود أي استفسار لديك يمكنك الاتصال ب:

الباحث الرئيسي: محمد جمال حسانين عطية	تليفون:01112760284

مقرر لجنة الأخلاقيات:....................................... تليفون:...............................................

---------------------------------------------------------------------------------------------------------------------

****أقر أنني اطلعت وفهمت الأجراءات التي ستتم من خلال هذا البحث ووافقت عليها**.

المشارك في البحث	الباحث الرئيسي

الأسم:........................................................ ................................

التوقيع (البصمه):........................................... ................................

التاريخ:......................................................

**ملحوظه: من حق المتطوع الحصول علي صورة من الاقرار.

هذا البحث:

توطئه لرسالة ماجستير	( **√** )

توطئه لرسالة دكتوراه	(	)

بحث غير ممول	( **√** )

مشروع بحثي ممول	(	)

تمت الموافقه علي هذا البحث من قبل لجنة أخلاقيات البحث العلمي بتاريخ:............................................

هذه الموافقه سارية حتي:.......................................................

رئيس اللجنة	خاتم اللجنة

-2-
